# Mutation in *clpC1* encoding an ATP-dependent ATPase involved in protein degradation is associated with pyrazinamide resistance in *Mycobacterium tuberculosis*

**DOI:** 10.1038/emi.2017.1

**Published:** 2017-02-15

**Authors:** Shuo Zhang, Jiazhen Chen, Wanliang Shi, Peng Cui, Jia Zhang, Sanghyun Cho, Wenhong Zhang, Ying Zhang

**Affiliations:** 1 Department of Molecular Microbiology and Immunology, Bloomberg School of Public Health, Johns Hopkins University, Baltimore, MD 21205, USA; 2 Shandong Medicinal Biotechnology Centre, Shandong Academy of Medical Sciences, Jinan 250062, China; 3 Department of Infectious Diseases, Huashan Hospital, Fudan University, Shanghai 200040, China; 4 College of Pharmacy, Institute for Tuberculosis Research, University of Illinois at Chicago, Chicago, IL 60612, USA

**Dear Editor,**

Pyrazinamide (PZA) is an important front-line tuberculosis (TB) drug used in combination with other TB drugs for the treatment of drug susceptible TB and MDR-TB.^1^ PZA has a critical role in shortening TB therapy^2^ because of its unique activity against *Mycobacterium tuberculosis* persisters that are not killed by other TB drugs.^1^ Despite the importance of PZA in shortening the treatment, its mechanism of action is the least understood of all TB drugs.^1, 2^ Mutation in the *pncA* gene encoding nicotinamidase/pyrazinamidase (PZase)^3^ involved in conversion of the prodrug PZA to the active form pyrazinoic acid (POA) is the major mechanism for PZA resistance in *M. tuberculosis.*^3, 4, 5^ A new target of PZA was identified as ribosomal protein S1 (RpsA) involved in trans-translation,^6^ which is involved in degradation of toxic protein buildup in stressed bacteria needed for persister survival. However, some low level PZA-resistant strains (MIC=200–300 μg/mL, pH 6) do not have mutations in either *pncA* or *rpsA* gene.^5, 7^ To identify new mechanisms of PZA resistance, we recently characterized a large number of *in vitro* *M. tuberculosis* mutants resistant to PZA and identified a third gene *panD* encoding aspartate decarboxylase as a new mechanism of PZA resistance and action.^8, 9^ In that study, in addition to *panD* mutations, we also found mutations in other genes such as *ppsA* encoding polyketide synthase involved in phthiocerol dimycocerosate (PDIM) synthesis, (3R)-hydroxyacyl-ACP dehydratase subunit HadC, acyl-CoA synthetase, cell division protein FtsH, phosphate ABC transporter permease protein PstC2, transmembrane transport protein MmpL4, putative transmembrane cation transporter and TetR family transcriptional regulator (Supplementary Table 1 of Zhang *et al.*^8^). However, we did not follow up on these candidate genes as all the mutant strains had *panD* mutations in addition to the above gene mutations. A recent study reported *ppsA-E* operon nonsense mutations were associated with PZA resistance based on whole-genome sequencing analysis of POA-resistant mutants^10^ using selection with high POA concentrations at neutral pH, a condition that was previously shown to allow POA-resistant mutants to be successfully isolated instead of conventional acid pH used for drug susceptibility testing.^9^ However, it is unclear how *ppsA-E* mutations cause PZA/POA resistance.

As we know there are other PZA-resistant strains such as strain 9739^(5)^ that do not have mutations in any of the known genes *pncA, rpsA* or *panD* involved in PZA resistance, in this study, to identify possible new mechanisms of PZA resistance, we characterized additional low level PZA-resistant *M. tuberculosis* H37Rv mutants (MIC=200, 300 μg/mL PZA, pH 60) identified in the previous study.^8^ By sequencing the known genes involved in PZA resistance including *pncA, rpsA* and *panD*, we were able to identify one low level PZA-resistant mutant S14 (MIC=200–300 μg/mL PZA, pH 6) that did not have any mutations in *pncA, rpsA* or *panD.* The genomic DNA from the PZA-resistant mutant S14 was isolated and subjected to whole-genome sequencing as described previously.^8^ Comparative sequence analysis of the PZA-resistant mutant S14 with the parent strain *M. tuberculosis* H37Rv as a reference revealed a single unique mutation of G to T change at nucleotide position 296 (G296T), causing amino acid change G99D in a new gene *clpC1* (Rv3596c), which encodes an ATP-dependent ATPase involved in protein degradation in conjunction with ClpP1 and ClpP2. Subsequent PCR was performed to amplify the *clpC1* gene from the PZA-resistant mutant S14 using forward primer F: 5′-ACG CTT GGG TGG TTT TCT CGT TTA TCT CCT ATG-3′, and reverse primer R: 5′-ACA AAC CGA CGT CAG CAG AGT CTA TTGTCA-3′. Sanger DNA sequencing of the *clpC1* PCR product verified the above mutation (G296T change, causing amino acid substitution G99D) identified by whole-genome sequencing to be genuine.

The *M. tuberculosis* ClpC1 is an 848-amino acid protein, which belongs to the large AAA family of ATPases. Owing to its essentiality in *M. tuberculosis*,^11^ ClpC1 has been proposed as a good drug target. ClpC1 is a hexamer that recognizes misfolded proteins and forms a complex with protease ClpP1 and ClpP2 to perform the function of protein degradation, a process that requires ATP hydrolysis by the ATPase activity of ClpC1.^11^ Interestingly, ClpC1 has recently been found to be a target of three new cyclic peptide antibiotics including cyclomarin A,^12^ lassomycin^13^ and ecumicin.^14^ The three new drug candidates appear to bind at the different sites of the ClpC1. For example, mutations of *clpC1* that caused resistance to lassomycin clustered at the N-terminal repeat region at Q17R, R21S and P79T.^13^ It is interesting to note that ecumicin-resistant mutations are located at L92S or F or L96P sites of the ClpC1 in *M. tuberculosis*,^14^ which are close to the G99D mutation in our PZA-resistant mutant S14. Because we found the PZA-resistant mutant S14 without *pncA, rpsA* and *panD* mutations had the ClpC1 G99D mutation (see above), we wanted to test if the two ecumicin-resistant strains with ClpC1 mutations L96P and L96F are resistant to PZA. Interestingly, we found that both L96P and L96F ClpC1 mutants were only marginally resistant to PZA (MIC=50 and 100 μg/mL PZA, respectively, pH 6, compared with susceptible control strain H37Rv, MIC<50 μg/ml), which were more susceptible to PZA than S14 with the ClpC1 mutation G99D (PZA MIC=200–300 μg/mL PZA, pH 6). This could indicate that certain sites such as G99D may be preferentially involved in POA binding and confer higher resistance than adjacent sites in L96P and L96F ClpC1 mutants. Further studies are required to confirm this possibility.

It is worth noting that all three new cyclic peptide antibiotics are active against both replicating and non-replicating mycobacteria, indicating ClpC1 could be an important drug target for both growing and persister bacteria. The anti-persister activity of the cyclic peptide antibiotics via their action on the protein degradation pathway is consistent with the previous observation that PZA, a well-known persister drug, shows its anti-persister activity at least partly through its inhibition of trans-translation,^6^ as part of the bacterial protein degradation systems. Future studies are needed to address if PZA alters ClpC1 activity thereby affecting protein degradation function of the ClpC1/ClpP1/ClpP2 complex leading to increased toxic protein buildup in *M. tuberculosis.*

To confirm that ClpC1 is a possible target of PZA, we cloned the *M. tuberculosis clpC1* gene and its promoter (174 bp upstream of the coding sequence) into pOLYG vector using the primers clpC1 F 5′-GCG AAG CTT TTT TCT CGT TTA TCT CCT ATG C-3′ and clpC1 R 5′-ACA GGA TCC CGT CAG CAG AGT CTA TTG TCA-3′. The pOLYG-*clpC1* and empty vector were electroporated into *M. tuberculosis* H37Ra and the transformants were tested for PZA susceptibility at 0, 25, 50, 100, 200 and 400 μg/mL PZA (pH 5.9). However, the pOLYG-*clpC1* behaved similarly as the pOLYG empty vector control as they did not grow at 50 μg/mL or higher concentrations of PZA, indicating overexpression of *clpC1* at least from its own promoter did not confer significant PZA resistance (data not shown).

While our study was ongoing, we noticed a recent study by Yee *et al.*^15^ who identified *clpC1* mutations from POA-resistant mutants that did not have *panD* mutations to be associated with POA resistance. Although we found the *clpC1* mutation in a single PZA-resistant mutant that did not have known PZA resistance mutations in *pncA, rpsA* and *panD* for some time,^8^ we withheld the publication of our *clpC1* results, largely because overexpression of the wild type *clpC1* in *M. tuberculosis* did not seem to confer significant resistance to PZA (MIC<50 μg/mL, pH 5.9; data not shown) and that we wanted to determine if ecumicin-resistant *clpC1* mutants are also resistant to PZA, which seemed to cause only marginal PZA resistance (see data above).

Identifying new mechanisms of PZA resistance using clinical isolates has been difficult because of the diverse genetic background of the clinical strains. Here, by using whole-genome sequencing of isogenic mutants resistant to PZA derived from the same strain H37Rv, we were able to identify a single mutation in the *clpC1* gene as a possible new mechanism of PZA resistance in addition to the known PZA resistance genes *pncA, rpsA* and *panD*. The mode of action of PZA is complex and it is becoming increasingly clear that PZA interferes with multiple targets involved in persister survival in *M. tuberculosis.*^1^ Future studies are needed to address the role of the identified *clpC1* mutations as a new mechanism of PZA resistance and confirm if ClpC1 serves as a possible new target of PZA in *M. tuberculosis.*
